# Current progress of plant-derived exosome-like nanovesicles on the regulation of osteoporosis and osteoarthritis

**DOI:** 10.1080/07853890.2025.2549524

**Published:** 2025-08-22

**Authors:** Xianting Xia, Jinguo Zhu, Xuexin Xu, Weiming Wang, Rui Zhao, Kai Wu, Haoqiang Huang, Yinhua Qian, Zhiwen Luo, Feng Xu, Zhijian Peng, Qing Wang

**Affiliations:** a Department of Orthopaedics, Kunshan Sixth People’s Hospital, Kunshan, Jiangsu, China; b Department of Orthopaedics, Nantong Tongzhou Hospital of Traditional Chinese Medicine, Tongzhou, Jiangsu, China; c Institute of Medicine, Wuhan University of Science and Technology, Wuhan, Hubei Province, China; d Department of Orthopaedics, Kunshan Hospital of Chinese Medicine, Kunshan, Jiangsu, China; e Department of Sports Medicine, Huashan Hospital, Fudan University, Shanghai, China

**Keywords:** Plant deprivative, exosome-like nanovesicles, osteoporosis, osteoarthritis, bone

## Abstract

Osteoporosis (OP) and osteoarthritis (OA) are clinically common chronic orthopedic diseases. With the aging of the global population, OP and OA pose a serious threat to human health. Exosomes are nanoscale vesicles secreted by cells and can mediate inter-cell communication. Recent research has shown that plant-derived exosomes-like nanovesicles (PELNs) can affect the proliferation and differentiation of osteoclasts, osteoblasts, bone marrow mesenchymal stem cells (BMSCs) and chondrocytes, regulate the immune system and inhibit inflammatory responses, and have potential value in treating OP and OA. This article summarizes the basic concepts, formation and components, separation and characterization methods of PELNs, and focuses on discussing the impact of PELNs on OP and OA, aiming to provide new ideas for the research of OP and OA.

## 1. Introduction

**Figure 1.**
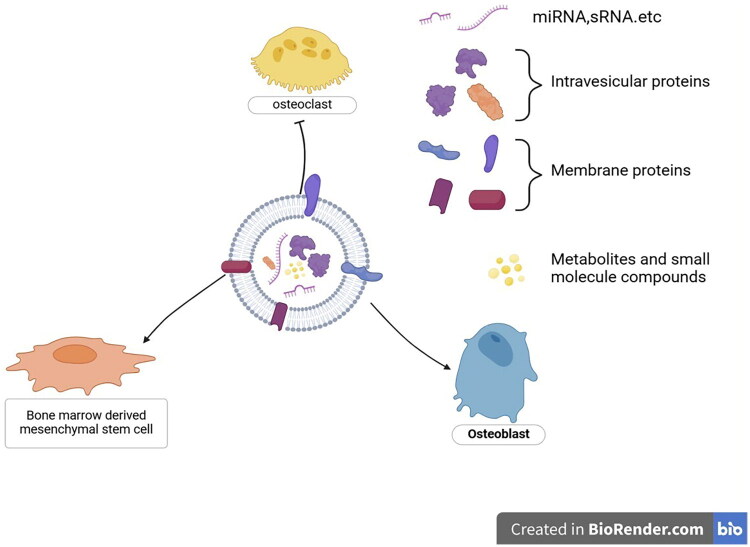
Schematic illustration of plant-derived exosome-like nanovesicles (PELNs) in osteoporosis and osteoarthritis regulation. PELNs modulate bone metabolism by inhibiting osteoclast differentiation via suppression of RANKL signaling pathways and promoting osteoblast proliferation/differentiation through BMP-2/Smad/RUNX2 pathways. In osteoporosis, PELNs enhance osteogenic differentiation of bone marrow mesenchymal stem cells (BMSCs) and reduce oxidative stress. For osteoarthritis, PELNs attenuate inflammation by inhibiting NF-κB activation and pro-inflammatory cytokines (e.g., TNF-α, IL-6), while promoting cartilage regeneration via delivery of growth factors and metabolic support to chondrocytes. Created with BioRender.com.

Osteoporosis is characterized by a reduction in bone mass, deterioration of microarchitecture, and increased fracture risk [1–3]. Epidemiological data indicate that osteoporosis affects one in three women and one in five men aged over 50, posing lifelong health burdens [4]. Osteoarthritis, the most prevalent form of arthritis among adults, is marked by chronic pain and loss of mobility. Its incidence significantly increases with age, particularly after the age of 40 [5]. Current OP management combines pharmacological interventions with adjunctive strategies: weight-bearing exercise and calcium/vitamin D supplementation remain foundational non-pharmacological approaches [6]. Conventional therapeutic agents include bisphosphonates, hormone replacement therapy (HRT), selective estrogen receptor modulators (SERMs), and calcitonin. In recent years, emerging pharmacological options such as fluoride compounds, parathyroid hormone analogs, and prostaglandin E2 derivatives have been introduced into clinical practice. Recent studies have further demonstrated the therapeutic potential of plant-derived exosome vesicles in osteoporosis management through modulation of bone metabolic homeostasis [7]. Exosomes are spherical nanovesicles, ranging from 30 to 100 nanometers in diameter, that are secreted by cells into the extracellular environment, facilitating intercellular communication [8–11] and the transport of biomolecules [12–15]. Previous exosome studies have focused on exosomes of animal cell origin, and in recent years, exosomes of plant origin have gradually been emphasized [14,16]. As a natural component, plant-derived exosome-like nanovesicles (PELNs) have been shown to possess structural and functional similarities to their mammalian counterparts [17]. Plant-derived exosome-like nanovesicles (PELNs) are more accessible and easier to produce at scale compared to animal exosomes. Due to their distinctive physiological, chemical, and biological properties, PELNs have significant potential for applications in disease therapy and the development of nanocarrier drug delivery systems (DDS) capable of delivering varying dosages [18–21]. Recent studies suggest that PELNs may hold promise for the treatment of osteoporosis and osteoarthritis through mechanisms involving the regulation of bone metabolism, reduction of chronic inflammation, and promotion of cartilage regeneration. In this review, we summarize the research advancements regarding the role of plant-derived exosome-like nanovesicles in the modulation of osteoporosis and osteoarthritis, aiming to provide novel insights for both clinical and fundamental research in these areas (Figure 1).

## 2. Biogenesis and components of exosome-like nanovesicles of plant origin

### 2.1. Biogenesis of PELNs

Current studies have identified three potential pathways for the biogenesis of plant-derived exosome-like nanovesicles (PELNs): the EXPO (exocyst-positive organelle) pathway, the multivesicular bodies (MVBs) pathway, and the vesicular pathway [22]. Among these, the MVBs pathway has been shown to closely resemble the biogenesis pathway of mammalian-derived exosomes and is the primary route for the formation of PELNs. The basic process involves the invagination and budding of the cytoplasmic membrane to form early endosomes, which further invaginate and mature into late endosomes that communicate with the Golgi network. Within these late endosomes, intraluminal vesicles (ILVs) containing cytoplasmic components, DNA, RNA, and lipids are formed. These ILVs are then transformed into ILV-containing MVBs, which are released into the extracellular space through fusion with the cytoplasmic membrane, thereby releasing ILVs into the extracellular environment to form PELNs [23]. The EXPO pathway refers to the intracellular formation of EXPOs with a double-membrane structure similar to that of autophagosomes; these EXPOs fuse with the plasma membrane and release single-membrane extracellular vesicles into the cell wall [24]. The vesicular pathway involves the fusion of cytosolic ILVs with the cytoplasmic membrane, resulting in the formation of vesicles that are released into the extracellular space [25].

### 2.2. Components of PELNs

The composition of plant-derived exosome-like nanovesicles (PELNs) is complex and influenced by various factors, including plant species and environmental conditions, encompassing multiple types of biomolecules [26,27]. These biomolecules primarily include: 1. Lipid bilayer [28]: The outer structure of PELNs consists of a lipid bilayer, which represents the fundamental structural feature. This lipid bilayer contains various lipids, such as phosphatidic acid, phosphatidic acid diamide, and phosphatidylcholine. These lipids and lipid assemblies may function as signals that facilitate preferential uptake by different receptor cells; 2. Proteins: The protein composition includes membrane proteins, cytoplasmic proteins, and specific proteins. Some studies suggest that membrane proteins may play a role in enhancing the uptake and internalization of PELNs by mammalian cells; however, there is a lack of evidence linking these proteins to the intrinsic therapeutic activity of PELNs; 3. Nucleic acids [29]: PELNs may contain various types of RNA, such as microRNAs (miRNAs) and small RNAs (sRNAs), which could be involved in regulating gene expression across species; 4. Metabolites and small molecule compounds: PELNs may include a range of metabolites and small molecule compounds that reflect the metabolic state of plant cells and may play a role in intercellular communication by transmitting signals.

## 3. Isolation and characterization of plant-derived exosome-like nanovesicles

The isolation of plant-derived exosome-like nanovesicles (PELNs) primarily relies on techniques such as ultracentrifugation [30], polymer precipitation [31], and size-exclusion chromatography [32]. Ultracentrifugation, currently the most widely standardized method, employs gradient centrifugation to remove impurities and enrich PELNs [33]. Polymer precipitation (e.g. using polyethylene glycol) achieves rapid sedimentation by competitively binding water molecules, offering advantages of operational simplicity and high-throughput capability [34]. Emerging technologies, including hydrophobic chromatography based on capillary-channeled polymer fibers, enable efficient isolation of PELNs from diverse fruits and vegetables, yielding 2.3 × 10^9 to 8.0 × 10^10 particles/mL [35,36]. Zhao et al. [37] have recently developed an enzymatic plant cell wall degradation-based method for extracellular vesicle (EV) isolation, which has been successfully applied to extract EVs from Morinda officinalis roots. This innovative approach demonstrates enhanced EV yield with significantly reduced contamination from intracellular components compared to conventional extraction techniques.

For characterization, transmission electron microscopy (TEM) reveals that PELNs typically exhibit spherical or cup-shaped morphologies, with particle sizes ranging from 40 to 200 nm. Their colloidal stability is supported by a negatively distributed zeta potential (approximately −30 mV) [38]. Chemical analyses, guided by the MISEV guidelines [39], validate vesicle integrity through detection of marker proteins such as heat shock protein 70 (HSP70) and glyceraldehyde-3-phosphate dehydrogenase (GAPDH) [40]. Lipidomic studies highlight the biological roles of membrane components like phosphatidic acid (PA) and phosphatidylcholine (PC) [41], while RNA sequencing identifies PELNs-derived miRNAs (e.g. from Panax notoginseng) capable of modulating 4,010 human genes [42].

The choice of isolation methods significantly impacts vesicle size, surface charge, and bioactivity [43]. To enhance purity, combinatorial approaches (e.g. ultracentrifugation coupled with size-exclusion chromatography) are often employed [44].

## 4. PELNs play a role in regulating bone metabolism

**Figure 2.**
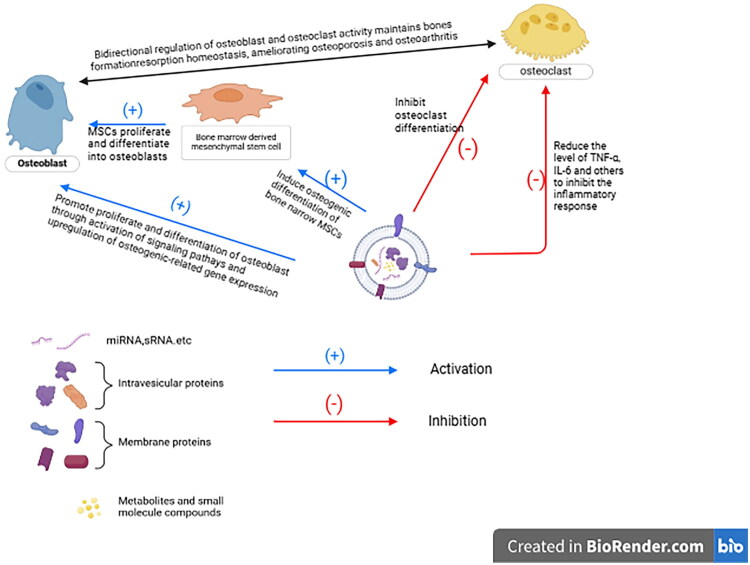
The regulatory role of PELNs on cells in bone metabolism.

Osteoporosis (OP) is a systemic bone metabolism disorder characterized by an increased risk of fragility fractures, which can lead to pain, functional impairment, and even mortality. Clinical pharmacological treatments for osteoporosis primarily include foundational bone metabolism supplements (e.g. calcium and vitamin D), bone resorption inhibitors (e.g. bisphosphonates, calcitonin), and bone formation enhancers (e.g. parathyroid hormone analogs) [45]. Mesenchymal stem cell (MSC)-derived exosomes modulate the immune system to regulate osteoclasts, osteoblasts, and osteoimmune processes [46]. For example: Adipose-derived MSC (ADSC) exosomes inhibit the NLRP3 inflammasome in osteoclasts of diabetic osteoporosis (OP) rat models and increase bone mineral density (BMD) [47]; MSC-derived exosomes enhance osteogenic differentiation of bone marrow-derived MSCs by suppressing M1 macrophage polarization and reducing levels of pro-inflammatory cytokines (e.g. IL-1β and IL-6) [48]. Although these medications demonstrate significant efficacy in reducing bone loss, increasing bone density, and lowering the incidence of osteoporotic fractures, they are also associated with certain adverse effects [49,50]. Recent studies have indicated that plant-derived extracellular nanovesicles (PELNs) can regulate bone metabolism and hold potential value for the treatment of osteoporosis, warranting closer attention (Figure 2).

### 4.1. PELNs inhibit osteoclast differentiation

An increased number of osteoclasts and enhanced bone resorption are important pathological mechanisms underlying the development of osteoporosis. Receptor activator of nuclear factor κB ligand (RANKL) is a key regulator of osteoclast maturation; it binds to the RANK receptor on osteoclast precursor cells to promote their differentiation [51–53]. Seo et al. [54] employed sucrose gradient centrifugation to isolate ginseng-derived extracellular nanovesicles (PELNs), which were found to contain high concentrations of the ginsenosides Rb1 and Rg1. Further studies revealed that ginseng-derived PELNs could inhibit RANKL-mediated signaling pathways and gene expression associated with osteoclast differentiation. Moreover, in animal experiments, these PELNs alleviated bone loss and improved bone microarchitecture in osteoporotic mice. Nanovesicles derived from plums also demonstrated the ability to enhance osteoblast differentiation while suppressing osteoclast activation [55].

### 4.2. PELNs promote the proliferation and differentiation of osteoblasts

Osteoblasts are regulated by various cytokines, and their primary function is the synthesis and mineralization of the bone matrix. Bone morphogenetic protein-2 (BMP-2), a member of the transforming growth factor-β superfamily, plays a crucial role in bone remodeling during adulthood. It promotes osteogenesis by binding to specific receptors and activating signaling pathways in osteoblasts [56]. Hwang et al. [57] found that yam-derived extracellular nanovesicles (PELNs) increased the gene expression of BMP-2 and stimulated the proliferation and differentiation of osteoblasts through the activation of the BMP-2/p-p38-dependent Runt-related transcription factor 2 (RUNX2) pathway. Similarly, a study by Sim et al. [58] demonstrated that apple-derived PELNs enhanced the expression of genes associated with osteoblast growth and differentiation, promoting osteogenesis by regulating the BMP2/Smad1 pathway. Furthermore, research by Park et al. [55] evealed that plum-derived PELNs exert bidirectional regulatory effects on bone, inhibiting osteoclast activity while promoting osteoblast differentiation. A recent study by Cao et al. [59] has demonstrated that MOEVLPs (Morinda officinalis-derived extracellular vesicle-like particles) significantly enhance proliferation of murine MC3T3-E1 cells (embryonic osteoprogenitor cells) and stimulate osteogenic activity, thereby promoting bone formation.

### 4.3. PELNs induce osteogenic differentiation of bone marrow MSCs

Bone marrow mesenchymal stem cells (BMSCs) are a class of pluripotent stem cells capable of differentiating into osteoblasts, adipocytes, chondrocytes, and other cell types [60]. It has been demonstrated that altered proliferation and differentiation of BMSCs serve as a primary cause of senile osteoporosis [61]. Furthermore, recent evidence indicates that cellular senescence of BMSCs also contributes to the development of senile osteoporosis [62,63]. The reduced osteogenic differentiation of BMSCs represents a crucial pathogenic mechanism underlying osteoporosis [64]. Zhao et al. [65] employed differential ultracentrifugation to extract PELNs and found that these vesicles could promote the osteogenic differentiation of BMSCs *via* the estrogen receptor α signaling pathway. Zhan et al. [66] reported that Clostridium perfringens in the feces of postmenopausal osteoporotic patients produces trimethylamine-N-oxide (TMAO), which inhibits the osteogenic differentiation of BMSCs as identified through sequencing. PELNs derived from Pueraria lobata can enhance the osteogenic differentiation of BMSCs by degrading TMAO. *In vitro* animal studies have further validated that Pueraria Mirifica-derived PELNs exhibit superior anti-osteoporotic effects. Oxidative stress is a significant pathological mechanism in the progression of osteoporosis, as it inhibits the proliferative capacity of BMSCs and results in decreased osteogenic differentiation alongside increased lipogenic differentiation of these cells. A study by Perut [67] found that strawberry-derived PELNs are rich in vitamin C, which mitigates oxidative stress and promotes the proliferation of BMSCs. Conversely, Gupta et al. [68] discovered that PELNs sourced from white powdery vine improved the oxidative stress status of BMSCs, enhanced their proliferation, and bolstered their osteogenic differentiation (Table 1).

**Table 1.** Recent advances and potential applications of plant-derived exosome-like nanoparticles (PELNs) in osteoporosis and osteoarthritis.

Author (Year)	Plant source	Disease model	Key findings	Proposed mechanism
Section 4: Osteoporosis		Osteoporosis		
Seo et al. (2023)	Ginseng	OP	Inhibited RANKL-mediated osteoclast differentiation; reduced bone loss in mice	Contains ginsenosides Rb1/Rg1; suppresses RANKL signaling pathways
Park et al. (2023)	Plum	OP	Enhanced osteoblast differentiation; suppressed osteoclast activation	Promotes BMP-2 expression and osteogenic markers (RUNX2, ALP, OCN)
Sim et al. (2023)	Apple	OP	Increased osteoblast differentiation and mineralization	Upregulated BMP-2/Smad1 pathway
Zhao et al. (2024)	Rhizoma Drynariae	OP	Reversed osteoporosis by enhancing osteogenic differentiation of BMSCs	Targeted ERα signaling pathway
Zhan et al. (2023)	Pueraria lobata (kudzu)	OP	Alleviated osteoporosis by enhancing autophagy in BMSCs	Activated autophagy via AMPK/mTOR pathway
Gupta et al. (2023)	Cissus quadrangularis	OP	Promoted osteogenic differentiation of BMSCs	Increased ALP activity and calcium deposition
Cao et al.(2024)	Morinda	OP	enhance proliferation of murine MC3T3-E1 cells	stimulate osteogenic activity, thereby promoting bone formation
Section 5: Osteoarthritis		Osteoarthritis		
Kim (2023)	Ginseng	OA	Reduced TNF-α and IL-6 levels; anti-inflammatory effects	Inhibited NF-κB activation
Zhu et al. (2023)	Garlic	OA/Colitis	Protected cartilage by modulating immune response	Suppressed TLR4/MyD88/NF-κB pathway
Yıldırım et al. (2024)	Tomato	OA	Enhanced chondrocyte markers (ACAN, SOX9, COMP); promoted cartilage regeneration	Delivered growth factors to chondrocytes
Chen et al. (2022)	Spinach	OA	Improved cartilage homeostasis under light exposure	Increased ATP/NADPH levels; enhanced chondrocyte anabolism
Qiu et al. (2020)	Curcumin (turmeric)	OA	Attenuated cartilage degradation and inflammation	Modulated miR-124/NF-κB and miR-143/ROCK1/TLR9 pathways
Perut et al. (2021)	Strawberry	OA	Protected chondrocytes from oxidative stress	Reduced ROS production; enhanced antioxidant defenses

This table compiles key studies evaluating PELNs from diverse plant sources in preclinical models of osteoporosis (OP) and osteoarthritis (OA). It highlights disease models, major findings, and proposed molecular mechanisms, including modulation of osteoblast/osteoclast activity, BMSC differentiation, anti-inflammatory effects, and chondrocyte regeneration. Studies are categorized by disease focus (OP or OA) and include references to experimental outcomes and signaling pathways.

## 5. Therapeutic effects of exosome-like nanovesicles of plant origin in osteoarthritis

Osteoarthritis (OA) is a prevalent degenerative disease affecting the bones and joints, primarily occurring in middle-aged and elderly individuals. The pathology of OA encompasses the entire joint, with lesions affecting the joint capsule, synovium, cartilage, subchondral bone, menisci, ligaments, and muscles. [69] These alterations result in localized pain, swelling, stiffness, and potential complications such as joint dysfunction and deformation [70,71]. OA exhibits multifaceted pathological mechanisms, including necrosis of synovial and chondrocyte cells, inflammatory responses, degradation of the extracellular matrix, cellular dysfunction, and impaired autophagy [72–75]. The pathogenesis of OA is complex and is primarily associated with factors such as age, genetics, trauma, obesity, and others. Currently, the clinical management of OA remains a significant challenge. Recent studies have indicated that PELNs possess the ability to regulate immune responses, inhibit inflammation, and promote soft tissue regeneration, which collectively may alleviate the symptoms of OA and warrant further investigation.

### 5.1. PELN inhibits the inflammatory response

The inflammatory response is a significant pathological mechanism in osteoarthritis (OA) [76–79]. It has been observed that certain PELNs exhibit excellent anti-inflammatory activity [80]. A study conducted by Kim [81] found that ginseng-derived PELNs could exert anti-inflammatory effects by inhibiting the activation of NF-κB, thereby reducing the levels of inflammatory factors such as tumor necrosis factor α (TNF-α) and interleukin 6 (IL-6). Zhu et al. [82] discovered that PELNs derived from garlic have the potential to protect the colon against DSS-induced damage by inhibiting the TLR4/MyD88/NF-κB signaling pathway and regulating gut microbiota. Vanessa’s study [83] demonstrated that goldberry-derived PELNs reduced the production of M1 macrophage products (e.g. NO) and promoted the polarization of M2 macrophages, thereby alleviating chronic inflammation in the body. Emmanuela’s research [84] indicated that PELNs sourced from Nigella sativa berries significantly decreased the expression of the IL-6 gene in cells, highlighting their important anti-inflammatory activity. Additionally, Iriawati [85] found that papaya-derived PELNs possess anti-inflammatory potential by upregulating the relative expression of anti-inflammatory cytokines such as IL-10 while downregulating pro-inflammatory cytokines such as IL-6 and IL-1β. A study by Wei [86] reported that turmeric-derived PELNs were enriched in curcumin and curcumin synthase, with the expression of curcumin synthase 2 being significantly higher than that in turmeric plant rhizomes. Curcumin has been shown to reduce the inflammatory response in OA and alleviate OA symptoms by inhibiting the activation of NF-κB, interleukin 8 (IL-8), nitric oxide synthase (NOS), prostaglandin E2 (PGE2), and cyclooxygenase-2 (COX-2) [87–90].

### 5.2. PELN promotes cartilage regeneration

Progressive degeneration and lysis of articular cartilage are hallmark features of osteoarthritis (OA) [91]. It has been found that miRNAs derived from plant-derived PELNs can regulate the plasticity of surviving cells at the injury site and modulate the extracellular matrix (ECM) to initiate cellular differentiation, proliferation, and tissue regeneration, thereby facilitating articular cartilage repair [92,93]. Yıldırım’s study [94] demonstrated that tomato-derived PELNs enhanced the expression of chondrocyte markers, including aggregated proteoglycan (ACAN), sex-determining region Y-framing protein 9 (SOX9), and cartilage oligomeric matrix protein (COMP), promoting cartilage regeneration. The study also revealed that PELNs could deliver growth factors to chondrocytes, contributing to the formation of a microenvironment conducive to cartilage regeneration and promoting the growth and maturation of new cartilage tissue. In a study by Chen et al. [95], after encapsulating spinach-derived nanocyst-like units (NTUs) with chondrocyte membranes (CM) and incorporating them into chondrocytes, it was found that CM-NTUs increased intracellular levels of ATP and nicotinamide adenine dinucleotide phosphate (NADPH), enhancing anabolic metabolism in degraded chondrocytes when exposed to natural light. This led to improved cartilage homeostasis and helped prevent the pathological progression of OA.

## 6. Quality assessment of existing recommendations

### 6.1. Current therapeutic frameworks and limitations

Current clinical guidelines for osteoporosis (OP) and osteoarthritis (OA) management rely on pharmacological and non-pharmacological interventions, yet exhibit significant limitations:

OP Therapies: Bisphosphonates (e.g. alendronate) and Selective Estrogen Receptor Modulators (SERMs; e.g. raloxifene) reduce fracture risk but carry long-term risks of atypical femoral fractures, osteonecrosis of the jaw, and gastrointestinal toxicity [45,49]. Parathyroid Hormone Analogs (e.g. teriparatide) promote bone formation but are limited by high cost, short-term use (≤24 months), and potential osteosarcoma risk [45]. Foundation Therapies (calcium/vitamin D) show modest efficacy in severe OP and poor adherence due to dosing frequency [6].

OA Therapies: NSAIDs (e.g. celecoxib) alleviate pain but accelerate cartilage degradation and pose renal/cardiovascular risks with chronic use [76,77]. Intra-articular Injections (corticosteroids/hyaluronic acid) offer transient symptom relief but may suppress cartilage matrix synthesis and fail to modify disease progression [73,75].

Most drugs lack tissue-targeted delivery, causing systemic side effects. Monotherapeutics inadequately address OP/OA complexity (e.g. OP involves osteoblast-osteoclast imbalance, inflammation, and oxidative stress [45,74]). Daily dosing regimens (e.g. bisphosphonates) reduce compliance [45] (Table 2).

**Table 2.** Limitations of current pharmacological interventions for osteoporosis and osteoarthritis.

Disease	Therapy category	Representative agents	Mechanism of action	Key limitations
OP	Bone Resorption Inhibitors	Bisphosphonates (e.g., Alendronate)	Induce osteoclast apoptosis	Atypical femoral fractures Osteonecrosis of jaw GI toxicity
	Selective Estrogen Modulators	Raloxifene	Bind to estrogen receptors	Increased thromboembolism risk, Limited efficacy in severe OP
	Bone Formation Promoters	Teriparatide (PTH analog)	Stimulate osteoblast activity	Osteosarcoma risk (animal models) ≤24-month use limit High cost
	Foundation Therapies	Calcium/Vitamin D	Support mineral homeostasis	Poor adherence (daily dosing) Minimal benefit in advanced OP
OA	Oral Symptom Modifiers	NSAIDs (e.g., Celecoxib)	Inhibit COX-1/2 enzymes	Accelerated cartilage degradation Renal/CV risks (↑30% with chronic use)
	Injectable Agents	Corticosteroids	Suppress synovial inflammation	Transient relief (≤4 weeks) Cartilage matrix suppression
		Hyaluronic Acid	Enhance joint lubrication	Limited structural improvement Variable efficacy across patients

### 6.2. PELNs as a strategic alternative to address limitations

PELNs demonstrate unique advantages over conventional therapies through multi-targeted actions:

Enhanced Safety Profile: Plant-derived lipids (e.g. phosphatidylcholine) confer biocompatibility without cytotoxic effects, contrasting with synthetic drug carriers [28,41]. Natural anti-inflammatory cargo (e.g. curcumin in turmeric PELNs) reduces OA inflammation without NSAID-related toxicity [86,88].

Multi-Pathway Modulation: For OP: Ginseng PELNs simultaneously suppress RANKL-induced osteoclastogenesis and activate BMP-2/RUNX2 osteogenic pathways, outperforming single-target drugs [54,57]. For OA: Tomato PELNs deliver growth factors (e.g. SOX9) to stimulate cartilage regeneration while inhibiting NF-κB-driven inflammation [94].

Surface proteins (e.g. HSP70) may enable selective bone homing, akin to animal exosomes [40,96], minimizing off-target effects (Table 2).

## 7. Conclusion

Osteoporosis (OP) and osteoarthritis (OA) are prevalent chronic skeletal disorders. Extensive studies have shown that traditional Chinese medicine (TCM) can regulate bone metabolism, reduce chronic inflammation in the body and promote cartilage regeneration, which is an effective treatment for osteoporosis and osteoarthritis, but the mechanism is still unclear. Structurally and functionally analogous to animal-derived exosomes, PELNs demonstrate broad regulatory effects on osteoclasts, osteoblasts, and bone marrow mesenchymal stem cells (BMSCs), enhance chondrocyte regeneration, and exhibit anti-inflammatory activity, suggesting their potential as a therapeutic mechanism of traditional Chinese medicine (TCM) in mitigating osteoporosis and delaying osteoarthritis progression. Notably, PELNs exhibit wider biological origins and superior scalability for large-scale production compared to animal exosomes.PELNs are similar to exosomes from animal cell sources in structure and function, and have a wider source and easier mass production than animal exosomes, which is expected to be a new approach for the treatment of osteoporosis and osteoarthritis in the future, but there are still a few issues that deserve attention.

Plant species, growth age, cultivation environment, and tissue sampling sites significantly influence PELNs properties [25]. Thus, standardized sourcing criteria for PELNs derived from identical plant species must be established. For functional studies of herbal PELNs, priority should be given to locally sourced herbs with defined growth environments, maturation periods, and consistent therapeutic efficacy to enhance experimental reproducibility. Additionally, PELNs characteristics are affected by isolation, purification, and characterization methodologies. A unified technical system for the separation and purification of PELNs should be established as soon as possible, and the specific markers of PELNs should be clarified. In addition, the medicinal properties of traditional Chinese medicine are affected by the method of concoction, and whether the method of concoction affects the function of extracellular vesicles secreted by plants of medicinal origin needs to be clarified by further research.

Similar to animal-derived exosomes, it is the contents of PELNs that play biological roles. Comprehensive lipidomic, transcriptomic, proteomic, and metabolomic profiling using multi-omics technologies with multi-parametric assessment is essential for identifying herbal vesicle biomarkers, evaluating PELNs’ therapeutic value, and facilitating translational applications. However, OP and OA pathological mechanisms are more complex, involving various factors such as aging, inflammation, and hormones. While existing studies suggest PELNs modulate osteoblast/chondrocyte differentiation and immune cell function, research has predominantly focused on phenotypic outcomes in bone metabolism and immunomodulation. The specific mechanisms underlying PELNs’ cross-species regulation of bone metabolism remain underexplored and require further elucidation.

Previous studies indicate that PELNs from different plant sources exert distinct biological effects. This warrants investigation into whether synergistic applications of multi-source PELNs, guided by TCM theory, could enhance efficacy while reducing toxicity. Finally, exosomal targeting capability is critical for maximizing therapeutic effects and minimizing adverse reactions. Although some animal-derived exosomes exhibit bone-targeting properties [96,97] and whether different PELNs have similar bone targeting properties needs to be confirmed by a large number of experiments. If highly bone-targeting PELNs can be identified, it is expected that PELNs will be utilized to develop new drugs with bone-targeting properties in the future.

## Data Availability

Data sharing is not applicable to this article as no new data were created or analyzed in this study.

## Literature screening methodology

The articles were selected with a narrative review.
